# Supplementary material for: Reversible tuning of magnetocaloric Ni-Mn-Ga-Co films on ferroelectric PMN-PT substrates
Source: Sci Rep. 2017 Oct 31;7:14462. doi: 10.1038/s41598-017-14525-3 (PMC5663903; doi:10.1038/s41598-017-14525-3)
Supplement: Supplementary file 1 — Supplementary information [file 41598_2017_14525_MOESM1_ESM.pdf]

Reversible tuning of magnetocaloric Ni-Mn-Ga-Co films on ferroelectric PMN-PT substrates

Benjamin Schleicher[1,2,*], Robert Niemann[1], Stefan Schwabe[1], Ruben Hühne[1], Ludwig Schultz[1,2], Kornelius Nielsch[1,2], and Sebastian Fähler[1,2]

[1]IFW Dresden, Institute for Metallic Materials, Helmholtzstr. 20, D-01069 Dresden, Germany
[2]TU Dresden, Faculty of Physics, D-01062 Dresden, Germany
[*]b.schleicher@ifw-dresden.de

ABSTRACT

This document contains the supplemental material for the above mentioned article.

1 Calculation of lattice parameters from reciprocal space maps (RSM)

Exemplarily, the RSM of the PMN-PT (013) and $(0\bar{1}3)$ as well as Ni-Mn-Ga-Co (026) and $(0\bar{2}6)$ reflections without an applied electric field are shown in fig. S1. To find the exact positions of the peak-maxima, the peaks were fitted using a 2D peak function V based on a simplified pseudo-Voigt profile. A linear combination of a 2d Gauß- (G) and a 2d Lorentzfunction (L) was used:

$$V(x,z) = A \cdot [(1-\eta) \cdot G(x,z) + \eta \cdot L(x,z)] \tag{S1}$$

with the peak amplitude A and η as the fraction of the Lorentzfunction in the linear combination. The complete terms are as follows:

$$G(x,z) = \exp(-(r_1 \cdot (x-x_0)^2 + 2s_1 \cdot ((x-x_0)(z-z_0)) + t_1 \cdot (z-z_0)^2)) \tag{S2}$$

$$L(x,z) = \frac{1}{(r_2 \cdot (x-x_0)^2 + 2s_2 \cdot ((x-x_0)(z-z_0)) + t_2 \cdot (z-z_0)^2 + 1)} \tag{S3}$$

with the abbreviations r_i, s_i and t_i for better readability:

$$r_1 = \frac{\cos^2 v}{2\sigma_x^2} + \frac{\sin^2 v}{2\sigma_z^2}; s_1 = -\frac{\sin(2v)}{4\sigma_x^2} + \frac{\sin(2v)}{4\sigma_z^2}; t_1 = \frac{\sin^2 v}{2\sigma_x^2} + \frac{\cos^2 v}{2\sigma_z^2} \tag{S4}$$

$$r_2 = \frac{\cos^2 v}{2w_x^2} + \frac{\sin^2 v}{2w_z^2}; s_2 = -\frac{\sin(2v)}{4w_x^2} + \frac{\sin(2v)}{4w_z^2}; t_2 = \frac{\sin^2 v}{2w_x^2} + \frac{\cos^2 v}{2w_z^2} \tag{S5}$$

In the xz-plane, the peaks are modelled as ellipses as seen by the elliptical expressions inserted into both functions. The major and minor axes of the ellipses are characterized by the peak shape in x- and z-direction, respectively, which itself is characterized by the standard deviations of the Gauß- (σ_x,σ_z) and Lorentzfunction (w_x,w_z). The whole peak can be rotated in-plane by an angle v to allow for the major and minor axes not to be aligned with the coordinate system. The peak position is then obtained from a least square minimization of the difference between the function $V(x,z)$ and the measured values. The background is assumed to be a plane in the examined area. The fit was performed with two peaks, to account for the Cu-$K_{\alpha1}$ and Cu-$K_{\alpha2}$ radiation with the distance between them included as a boundary condition.

From the exact position of the Cu-$K_{\alpha1}$ peak obtained from the fitting procedure, the q_x and q_z values in the reciprocal space were calculated using the wavelength $\lambda_{\text{Cu-K}_{\alpha1}} = 0.15406\,\text{nm}$. In the following, q_i^+ and q_i^- refer to the q_i ($i = x,z$) of the (hkl)

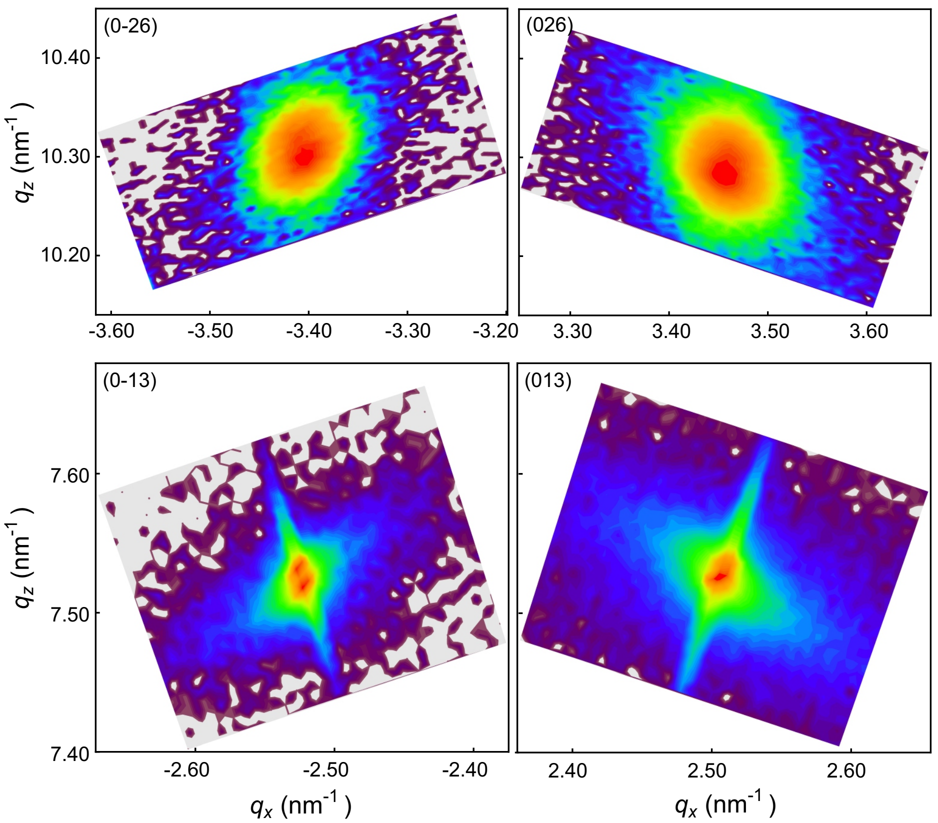

Figure S 1. Reciprocal space maps of the PMN-PT (013) and (0$\bar{1}$3) as well as Ni-Mn-Ga-Co (026) and (0$\bar{2}$6) reflections. For visibility, the axes were broken to enlarge the reflections.

and ($h\bar{k}l$) reflection, respectively and $\Delta q_i = q_i^+ - q_i^-$. From geometric considerations, the in-plane and out-of-plane lattice parameters a and c, respectively, were then calculated using the equations

$$a = \frac{2k}{\sqrt{\Delta q_x^2 + \Delta q_z^2}}; c = \frac{l}{\sqrt{((q_x^+)^2 + (q_z^+)^2) - (0.25 \cdot (\Delta q_x^2 + \Delta q_z^2))}}\,. \tag{S6}$$

2 Magnetization measurements at 2 T

Figure S2 shows $M(T)$ measurements at $\mu_0 H = 2\,\mathrm{T}$ for two different electric fields $E = 0\,\mathrm{kV/cm}$ and $E = 3\,\mathrm{kV/cm}$. The changes of the magnetization due to an applied electric field are the same as for a magnetic field of $\mu_0 H = 0.1\,\mathrm{T}$ shown in the main article. There is no shift of the transition temperature, but in the austenite region (fig. S2(b)) there is an increase of the magnetization as well as a shift of the local magnetization maximum to lower temperatures. At this high magnetic field, the sample is magnetically saturated (see fig. S3) and the influence of the mechanical stress on the magnetization in the austenite is the same as for $\mu_0 H = 0.1\,\mathrm{T}$. It can therefore be concluded that magnetostriction is not responsible for the observed changes of the magnetization but a change of spontaneous magnetization.

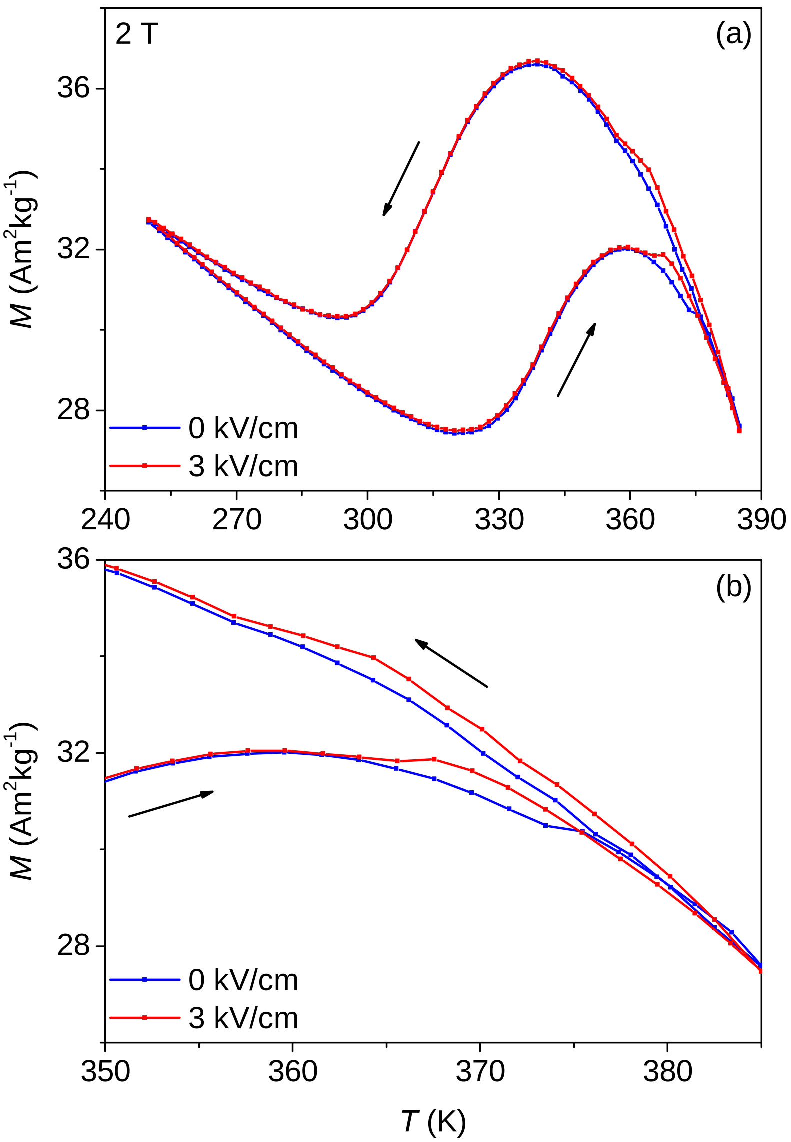

Figure S 2. (a) $M(T)$ measurements for $250 \leq T \leq 385\,\mathrm{K}$ for two different electric fields at $\mu_0 H = 2\,\mathrm{T}$. Similar to measurements at $0.1\,\mathrm{T}$, the main influence of the electric field on the magnetization was observed in the austenite region, where an increase of M is visible in the zoom-in (b).

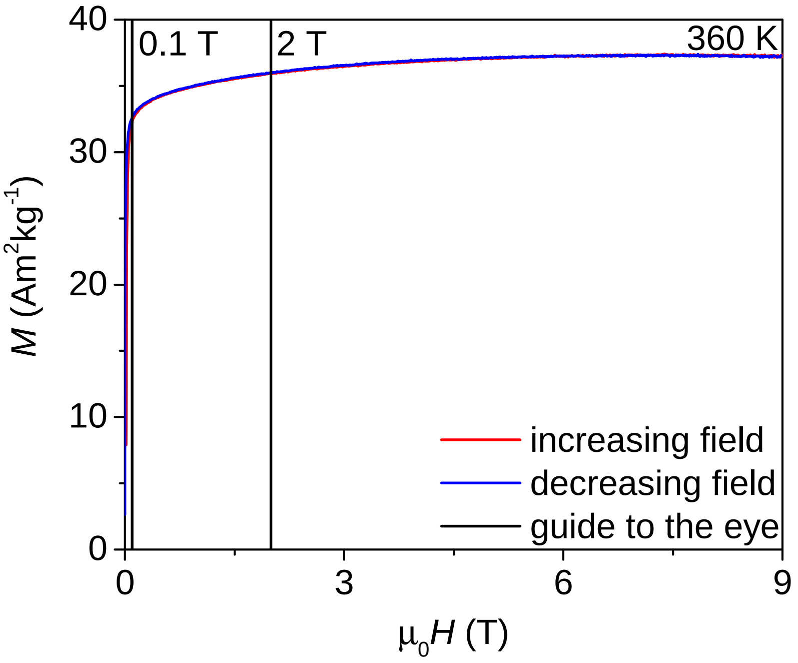

Figure S 3. $M(H)$-measurement in the austenite phase at 360 K. The black lines are guides to the eye for 0.1 T and 2 T. At 2 T, the sample is saturated and the $M(E)$-measurements at 0.1 T were performed close to saturation.
